# The Mechanics of Defæcation

**Published:** 1897-11-13

**Authors:** 


### THE MECHANICS OF DEFÆCATION.

In his Lectures on the Action of Medicines, a work by the way which is full of information and suggestions, Dr. Lauder Brunton devotes a page or two to a subject which is often too little considered in discussing the treatment of constipation, viz., the mechanics of defæcation. He points out that in the construction of mankind there is a double provision for preventing involuntary evacuation. First, there is the sigmoid flexure, which acts as a syphon trap, and prevents fæcal matters coming straight down from the descending colon into the rectum. The block here is not merely a matter of mechanics but of nervous relationship, for so long as the fæces lodge in the sigmoid they do not excite that peristaltic and reflex action which leads to defæcation, but whenever they get into the rectum this action is set up and a tendency to defæcate occurs.

Then, in consequence of the form of the pelvis, much as happens to the child's head during labour, the fæcal matters are pressed backwards during defæcation in such a direction that they do not tend to strike the anus, but a point a good deal behind it. Now this must be borne in mind, that the turning of the fæcal mass forwards towards the anus depends on the tension of the floor of the pelvis; and that occasionally, especially in women who have borne a good many children, and whose pelvic floor is very lax, the perineum may require to be supported just as it does in parturition. "In some patients who find that they cannot readily pass a motion, a little pressure just below the coccyx is sufficient to turn the fæcal mass forward, and allow them to get rid of it."

There is another way, however, in which the difficulty can sometimes be got over, viz., by the patient adopting such an attitude as shall give the requisite tension to the pelvic floor. Nearly all nations except the English and the Americans adopt the crouching attitude in defæcation, and some people who are quite unable to evacuate the bowels when sitting on a closet of the ordinary form are able to do so easily enough by crouching down, as is done in Germany, France, and elsewhere. "This may be done," says Dr. Brunton, "either by telling the patients to have in the closet a chamber-pot, *over* which, not *upon* which, they may sit, and thus evacuate the bowels. This seems a very simple thing to tell you, yet, oddly enough, I think one of the most grateful letters I ever had in my life was from a patient to whom I had given this simple instruction."

As bearing upon this point we may draw attention to a paragraph in Dr. Vivian Poore's "Essays on Rural Hygiene," in which he points out that the usual closet seat is too high, and that a much lower seat has "certain physiological advantages," which, however, will no doubt be still more effectually obtained by the plan suggested by Dr. Lauder Brunton.

